# Characterization of viral infections in children with influenza-like-illness during December 2018–January 2019

**DOI:** 10.3389/fcimb.2023.1351814

**Published:** 2024-01-18

**Authors:** Shuang Chao, Yuejiao Wang, Baolei Wu, Runqing Li, Jingxiao Dong, Lina Ji, Xuejun Li, Ran Li, Xiaomei Yin, Xiuying Zhao, Wannian Liang

**Affiliations:** ^1^ Department of Pediatrics, Beijing Tsinghua Changgung Hospital, School of Clinical Medicine, Tsinghua University, Beijing, China; ^2^ Vanke School of Public Health, Tsinghua University, Beijing, China; ^3^ Department of Clinical Laboratory, Beijing Tsinghua Changgung Hospital, School of Clinical Medicine, Tsinghua University, Beijing, China

**Keywords:** respiratory viral infection (RVI), influenza (flu), nucleic acid sequence-based amplification (NASBA), influenza rapid diagnostic test (IRDT), influenza-like illness (ILI), pediatrics

## Abstract

**Introduction:**

Respiratory viral infection (RVI) is of very concern after the outbreak of COVID-19, especially in pediatric departments. Learning pathogen spectrum of RVI in children previous the epidemic of COVID-19 could provide another perspective for understanding RVI under current situation and help to prepare for the post COVID-19 infection control.

**Methods:**

A nucleic acid sequence-based amplification (NASBA) assay, with 19 pairs of primers targeting various respiratory viruses, was used for multi-pathogen screening of viral infections in children presenting influenza-like illness (ILI) symptoms. Children with ILI at the outpatient department of Beijing Tsinghua Changgung Hospital during the influenza epidemic from 12/2018 to 01/2019 were included. Throat swabs were obtained for both the influenza rapid diagnostic test (IRDT) based on the colloidal gold immunochromatographic assay and the NASBA assay, targeting various respiratory viruses with an integrated chip technology.

**Results and discussion:**

Of 519 patients, 430 (82.9%) were positive in the NASBA assay. The predominant viral pathogens were influenza A H1N1 pdm1/2009 (pH1N1) (48.4%) and influenza A (H3N2) (18.1%), followed by human metapneumovirus (hMPV) (8.8%) and respiratory syncytial virus (RSV) (6.1%). Of the 320 cases identified with influenza A by NASBA, only 128 (40.0%) were positive in the IRDT. The IRDT missed pH1N1 significantly more frequently than A (H3N2) (P<0.01). Influenza A pH1N1 and A (H3N2) were the major pathogens in <6 years and 6-15 years old individuals respectively (P<0.05). In summary, influenza viruses were the major pathogens in children with ILI during the 2018-2019 winter influenza epidemic, while hMPV and RSV were non-negligible. The coexistence of multiple pathogen leading to respiratory infections is the normalcy in winter ILI cases.

## Introduction

Currently, respiratory infections caused by immune debt post the spreading of COVID-19 have attracted attention from both academic and medical communities (Principi et al., 2023). Seasonal respiratory viral infections (RVI) mainly affect the nose, throat, and airways, bring to nasal congestion, a runny nose, sore throat, cough, and to extent with fever, generally with influenza-like illness (Lafond et al., 2016). Numerous viruses, including influenza virus A (FluA) sub-strains A H1N1 pdm1/2009 (pH1N1), A (H3N2), and two lineages of influenza virus B (FluB) have been implicated in the pathogen spectrum of pediatric RVI (Paul Glezen et al., 2013; Bedford et al., 2015). Patients with RVI may develop severe symptoms or even life-threatening complications, particularly children, the elderly, and individuals with underlying chronic conditions (Al-Awaidy et al., 2015; Kwong et al., 2018). Upon introduction in poultry, other members of the Orthomyxoviridae family, such as the subtypes of H5 and H7, may lead to outbreaks of highly pathogenic avian influenza, or even the acute respiratory distress syndrome (ARDS) (Tang et al., 2019; Zhong et al., 2019). On the other hand, pathogenic microorganisms other than influenza viruses, like the respiratory syncytial virus (RSV) (Takashita et al., 2021); the members of the coronavirus family including the novel SARS-CoV-2, the human rhinovirus (HRV), adenoviruses (ADV), and mycoplasma pneumonia (MP), can contribute to influenza-like illness (ILI) symptoms (Adam et al., 2017); leading to confusion in both diagnosis and disease managing. After emergence of SARS-CoV-2, from the early spring of 2020 to late 2021, reported influenza cases declined considerably due to implementation of quarantine and improved hygiene behavioral changes (Cowling et al., 2020; Feng et al., 2021). While increases in influenza activity were observed with the FluB/Victoria lineage as major pathogen in the beginning of 2022. Cocirculation of SARS-CoV-2 with influenza viruses and other else co-infections has been paid more attention in winter of 2023 (Wolters et al., 2021; Yun et al., 2021).

According to recent guidelines (General Office of National Health Commission of People's Republic of China, 2018; Uyeki et al., 2019b), administration of oseltamivir is recommended for selected patients with severe influenza. Therefore, timely differentiation of influenza viruses, as well as other respiratory pathogens, is essential for clinical management. Influenza rapid diagnostic tests (IRDTs) targeting the antigens of FluA and FluB viruses are widely used for timely diagnosis (Chartrand et al., 2012). Unfortunately, given the high false-negative rates of IRDTs (Uyeki et al., 2009), they were removed from priority detection tools in recent guidelines (Miller et al., 2015). Instead, nucleic acid-based tests including real-time RT-PCR are recommended to assist in the diagnosis of respiratory viral infection (Uyeki et al., 2019a; Losier and Dela Cruz, 2022). Respiratory panel assays based on automated multiplex RT-PCR method or other isothermal amplification techniques that integrates all reagents required for common respiratory viruses have been promoted (Couturier et al., 2013; Takashita et al., 2021). One of them featured by detecting 17 respiratory viruses and 3 atypical pathogens, had been proved to be helpful in the diagnosis of acute respiratory infections (Loeffelholz et al., 2011), with good performance in the diagnosis of community-acquired pneumonia (CAP) and hospital-acquired infections (Busson et al., 2019; Thongpan et al., 2019). A high-throughput, multi-index isothermal amplification platform for rapid detection of 19 types of common respiratory viruses or subspecies, including SARS-CoV-2 had been reported in 2020, the assays based on nucleic acid sequence-based amplification (NASBA) and previously reported micro/nanofluidic chip platform (MNCP) (Xing et al., 2020). Other multiple assay platforms based on real-time nucleic acid assay have been incorporated into routine diagnosis during and post the COVID-19 epidemic, broaden the understanding of multiple viral pathogenic infections (Li et al., 2019).

Under such background, to clarify the pathogenic characteristics previous the COVID-19 pandemic would help us prepare for the post-COVID-19 era. The present study concerns on a NASBA platform and 19 pairs of primers for multiple respiratory viruses, focus on its application in screening and identifying viral infections in pediatric outpatients presenting ILI symptoms during the winter of 2018-2019. The results were summarized based on demographic and clinical characterization.

## Materials and methods

### Study population and sample collection

The study population included outpatients who sought medical attention at the Department of Pediatrics of Beijing Tsinghua Changgung Hospital during a severe influenza epidemic from December 2018 to January 2019. All patients were children aged 0-15 years whose cases met the standard definition of ILI, including sudden onset of fever (≥38°C) and a cough and/or a sore throat without a known cause other than influenza (National Health and Family Planning Commission of the People's Republic of the China, 2018). They were classified into four age groups: babies ≤12 months (≤12m, infant period), children >12 months but <3 years (12m-3y, toddler period), children ≥3 years but <6 years (3-6y, preschool age), and children ≥6 years but ≤15 years (6-15y, school age and adolescence) (**Table 1**). Two throat swabs were obtained from each patient, one of which was used for immediate IRDT and the other was stored in 500 µl of the viral transport medium (VTM) at -80°C for RNA extraction within 7 days.

**Table 1:** Characteristics of the outpatients included in the study.

Age groups	Cases (%)	Sex		Time since symptoms onset to diagnosis (days)	Pneumonia cases
		Male	Female		
≤12m	19 (3.7%)	11	8	2.0 ± 1.7	3
12m-3y	210 (40.5%)	107	103	2.0 ± 1.8	21
3-6y	191 (36.8%)	96	95	2.2 ± 2.0	10
6-15y	99 (19.1%)	55	44	1.5 ± 0.9	1

### Swab processing and RNA extraction

The throat swabs were vortexed for 30 s in the VTM (Yocon Biological Pharmacy, Beijing, China). Then, 300 μl of the medium was used for RNA extraction with TRIzol™ LS Reagent (Life Technologies Co., Grand Island, NY, USA) as recommended by the manufacturer. The RNA solution was obtained by adding 50 μl of RNase-free H_2_O.

### NASBA assay

The NASBA assay is an isothermal reaction coupled with the micro/nanofluidic chip (MNCP) platform. The data were analyzed with the software coupled with the RTisochip-A detector (CapitalBio Corp., Hong Kong, China) (Zhang et al., 2018). Briefly, 19.5 µl of RNA solution was mixed with amplification reagents, and the mixture was loaded into an MNCP, in which 24 reaction chambers are designed, with each chamber connected to a sine-shaped infusing channel by a micro-channel. The 19 pairs of primers targeted to the Flu A matrix protein (MP) gene, pH1N1 hemagglutinin (HA) gene, seasonal A H1N1 (sH1N1) HA gene, Flu A H3 HA gene, Flu A H7 HA gene (Chen et al., 2019), Flu B MP gene for general influenza B virus, RSV fusion protein (FP) gene, human entero-rhinovirus (HRV) 5’-UTR, enterovirus EV71 subtype (EV71) VP gene, coxsackievirus CA16 subtype (CA16) VP gene, coxsackievirus CA6 subtype (CA6) VP gene, ADV terminal protein gene, parainfluenza-1 (PIV1) hemagglutinin-neuraminidase (HN) gene, PIV2 HN gene, PIV3 HN gene, PIV4 HN gene, human metapneumovirus (hMPV) fusion protein (F) gene, universal primers for coronavirus OC43 and HKU1 (OC43/HKU1) orf1ab polyprotein gene, and universal primers for coronavirus NL63 and 229E (NL63/229E) orf1ab polyprotein gene. Totally 18 pathogens, the specific viruses causing respiratory symptoms, or its sub-species were included.

### Quality assurance and results interpretation

The system had been validated before it being used for the assay. To evaluate the sensitivity of the platform, the synthesized RNA templates were used to make sure that the sensitivity are below 200 copies/ul for all the pathogens. RNA from pH1N1 was used for the system repeatability test at concentration of 500 copies/µL (Xing et al., 2020). As a qualitative test, two positive controls, two negative controls were included. Assessment of the internal control (IC) glyceraldehyde 3-phosphate dehydrogenase (GAPDH) confirmed that swab samples were collected successfully, and nucleic acid extraction performed correctly. Only results with adequate positive, negative, and IC controls were used for analysis. The subtypes of Flu A sH1N1, pH1N1, A (H3N2), and A (H7N9) were identified with the specific influenza HA gene being positive. Influenza A (IA) was identified with only the Flu A MP gene being positive but the specific Flu A HA genes were negative.

### Influenza rapid diagnostic test

The Clearview Exact Influenza A&B by Alear (Abon Biopharm Co., Ltd., Shanghai, China) was used at Beijing Tsinghua Changgung Hospital for influenza antigen detection. The reagent is coated with anti-influenza A and anti-influenza B NP in one strip, enabling differentiation between Flu A and Flu B. Undiluted swabs from the 519 patients were used for detection immediately after collection. All specimens were tested in a single experiment according to the manufacturer’s instructions.

### Automatic blood cell analysis

Whole blood samples collected with EDTA.2K anticoagulant were tested for the 519 cases on a Sysmex XN1000 automatic blood cell analyzer (Sysmex Corp., Kobe, Japan).

### Statistical analysis

The data were analyzed with SPSS 24.0 for Windows (IBM, Armonk, NY, USA). Continuous variables with normal distribution (Kolmogorov-Smirnov test) were presented as mean ± standard deviations (SD) and analyzed by analysis of variance; otherwise, continuous data were presented as median (interquartile ranges) and analyzed by the Wilcoxon test. Categorical variables were presented as frequency (percentage) and analyzed by the chi-square test or Fisher’s exact test. Two-sided (except for the chi-square test) P<0.05 was considered statistically significant.

## Results

### Patient characteristics and general assay results

The study population included 519 outpatients (269 males and 250 females; 4.4 ± 3.2 years) who sought medical attention during the severe influenza epidemic of 2018-2019 (**Table 1**).

Of the 519 patients, 430 (82.9%) were tested positive for at least one pathogen, and the remaining 89 (17.2%) cases were tested negative. In the 430 positive cases, the predominant single virus infection was due to Flu A (293/430, 68.1%), including 208 (48.4%) cases of pH1N1 and 78 (18.1%) of A (H3N2), followed by hMPV (38/430, 8.8%), RSV (26/430, 6.1%), and OC43/HKU1 (13/430, 3.0%). The results indicated that Flu A mainly accounted for the ILI symptoms during this epidemic season. Cases with two or more pathogens were defined as co-infection cases (41/430, 9.5%), of which the most common combination was pH1N1 with RSV (10/430, 2.3%) (**Table 2**).

**Table 2:** Pathogen distribution in the 430 positive cases by the NASBA assay.

Pathogens	Subtypes	Coinfections	Positive N (%)	
Influenza A	pH1N1	/	208	48.4
	A(H3N2)	/	78	18.1
	IA	/	7	1.6
hMPV		/	38	8.8
RSV		/	26	6.1
OC43/HKU1		/	13	3.0
PIV1		/	5	1.2
HRV		/	4	0.9
Influenza B		/	3	0.7
PIV3		/	2	0.5
EV71		/	2	0.5
PIV4		/	1	0.2
Influenza A virus + Others	pH1N1	+ A(H3N2)	2	0.5
		+RSV	10	2.3
		+hMPV	4	0.9
		+HRV	3	0.7
		+NL	2	0.5
		+PIV1	2	0.5
		+PIV3	1	0.2
		+HRV+hMPV	1	0.2
		+ HRV+PIV2	1	0.2
	A(H3N2)	+RSV	5	1.2
		+OC43//HKU1	2	0.5
		+hMPV	1	0.2
		+HRV	2	0.5
	IA	+ADV	1	0.2
HRV		+hMPV	3	0.7
RSV		+CA16	1	0.2
		+hMPV	1	0.2
		+ADV	1	0.2
Total			430	100

### Virus identification in the different age groups

Children aged 3-6y (161/191, 84.3%) and 12m-3y (173/210, 82.4%) represented the majority of the viral positive cases, followed by the 6-15y (81/99, 81.8%) and ≤12m (15/19, 79.0%) groups; the frequency of the general positivity showed no significant difference among the tested age groups (χ2 = 0.82, P=0.84).

**Figure 1:**
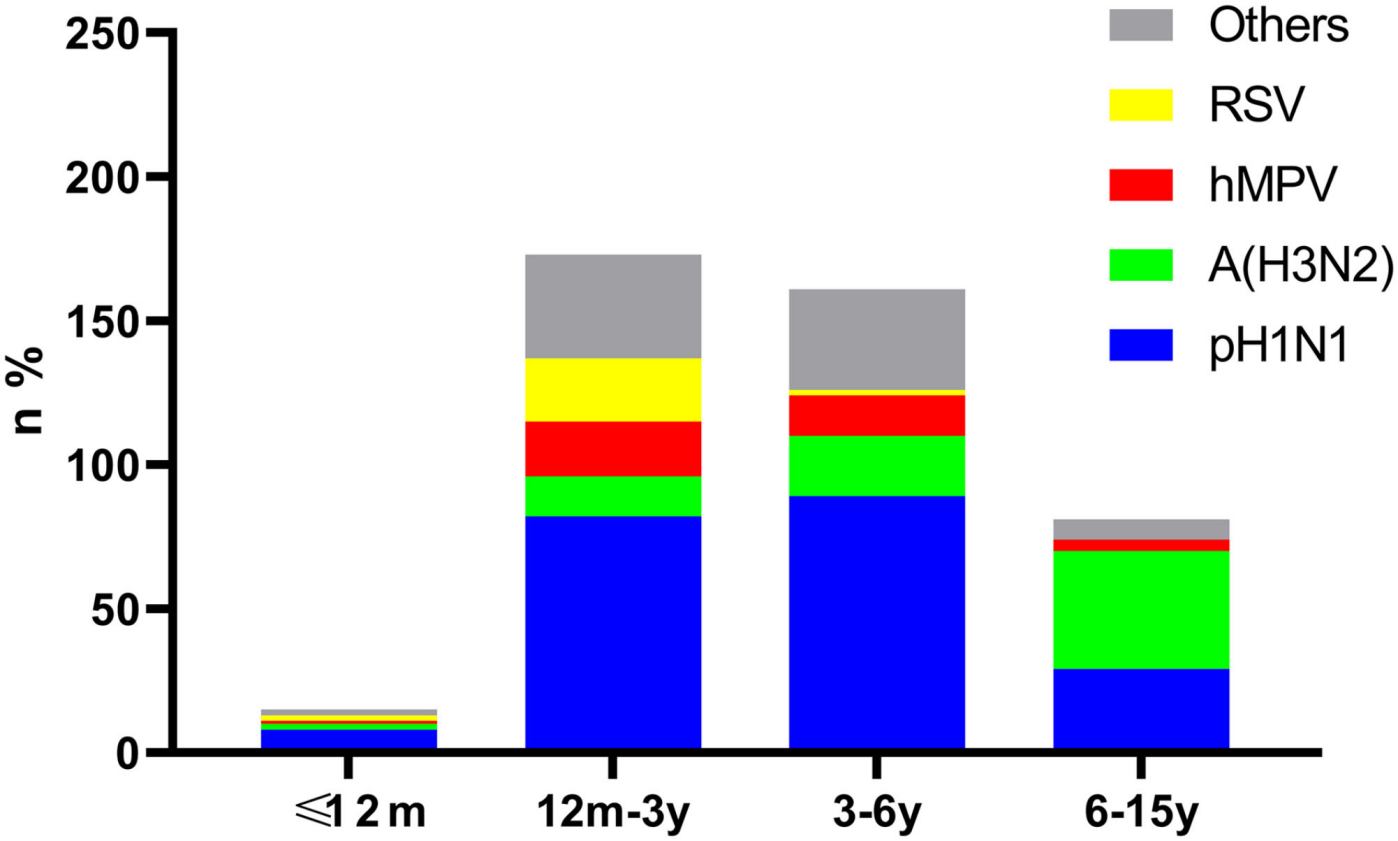
Age distribution of cases positive for pH1N1, A (H3N2), hMPV and RSV. Bars show the age distribution of case numbers and rates for pH1N1, H3N2, hMPV, RSV and other viruses in the 430 positive cases.

The detection rate was significantly different among age groups for pH1N1, A (H3N2) and RSV. Among the 430 positive cases, pH1N1 was the major pathogen in the 12m-3y and 3-6y groups, showing a significant difference between the 3-6y and 6-15y groups (χ2 = 8.18, P<0.001). Compared with the other groups, children aged 6-15y were mainly infected by A (H3N2) (P=0.01, <0.001, <0.001, respectively). Children aged 12m-3y were found to be infected with RSV more frequently than other viruses, differences between ≤12m vs. 3-6y, ≤12m vs. 6-15y, 12m-3y vs. 3-6y, and 12m-3y vs. 6-15y were statistically significant (P=0.04, 0.02, <0.001, and <0.001, respectively). Meanwhile, hMPV was mainly identified in the 12m-3y and 3-6y groups, but there were no significant differences among groups (P>0.05) (**Figure 1**).

### NASBA versus IRDT for influenza detection

There were 330 positive cases of Flu A (76.7%, 330/430) and 3 positive cases of Flu B (0.7%, 3/430). Sub-typing of Flu A indicated that 232 (232/330, 70.3%) were pH1N1, 88 (88/330, 26.7%) were A (H3N2), 8 (8/330, 2.4%) with IA positive were classified to Flu A group but could not be differentiated, and 2 (2/330, 0.6%) were co-infected by pH1N1 and A (H3N2).

Considering that the IRDT is still commonly used as a point-of-care test (POCT) for influenza diagnosis, we compared the results in 320 individuals positive for Flu A by the NASBA (232 tested positive for pH1N1, 88 tested positive for A (H3N2)) with IRDT results. In general, 128 of the 320 cases were positive for influenza A by the IRDT (40.0%). When analysis was performed by subtypes, the IRDT’s positivity rate in patients with pH1N1 was 27.6% (64/232), and that of patients with A (H3N2) was 72.7% (64/88). Compared with NASBA, the detection of A (H3N2) infection by the IRDT was significantly more efficient than that of pH1N1 infection (χ2 = 54.17, P<0.001) (**Table 3**). These results strongly suggested that the IRDT was likely to miss Flu A, especially as the epidemic was caused by the pH1N1 virus. From the perception, NASBA and other nucleic based assays could significantly improve the efficiency of influenza diagnosis versus IRDTs, especially when infections are caused by pH1N1.

**Table 3:** IRDT results in the pH1N1 and A(H3N2) infected groups.

Influenza A by NASBA	Positive by IRDT*	Negative by IRDT	Total
pH1N1	64 (27.6%)	168	232
A(H3N2)	64 (72.7%)	24	88
Total	128	192	320

*Totally 27.60% of pH1N1 cases and 72.70% of A(H3N2) cases tested positive by the IRDT. A significant difference was observed between Flu A subtypes and IRDT positivity (P<0.01).

### Clinical characteristics by virus infection

We compared white blood cell (WBC) counts, neutrophil (NE) counts, thermal spikes, frequencies of cough, and frequencies of other clinical characteristics in the four virus-positive groups with total cases number more than 25. Individuals who were hMPV-positive and RSV-positive were more likely to present with higher WBC counts compared with patients infected by pH1N1 or A(H3N2) (P-values were <0.001, 0.003, <0.001, and 0.006, respectively). No significant differences in WBC counts were found between pH1N1 and A (H3N2) cases (P=0.93), or between hMPV and RSV cases (P=0.55). In addition, children infected by hMPV tended to show higher NE amounts compared with those infected by the remaining three viruses (P=0.01, 0.01, 0.02, respectively). NE counts in children infected by pH1N1, A (H3N2) and RSV showed no significant differences (all P>0.05). There were significant differences in frequency of cough between A (H1N1)-positive (79.8%) and hMPV-positive (94.7%) individuals (χ2 = 4.88, P=0.03) (**Table 4**).

**Table 4:** Clinical characteristics of pH1N1, A(H3N2), hMPV and RSV infections.

	pH1N1 (n=208)	A (H3N2) (n=78)	hMPV (n=38)	RSV (n=26)
WBC (×10^9^/L) Median (range)	5.89 (2.12-15.84)	6.01 (2.50-14.98)	7.85 (2.95-19.42)^**^	7.41 (4.51-17.33)^**^
NE (×10^9^/L) Median (range)	3.50 (0.36-13.43)	3.78 (0.89-9.85)	4.09 (0.90-14.90)^*^	3.00 (1.42-10.06)
Cough, n (%)	166 (79.8%)	66 (84.6%)	36 (94.7%)^*^	24 (92.3%)
Pneumonia, n (%)	14 (6.7%)	3 (3.9%)	3 (7.9%)	4 (15.4%)
Vomiting or diarrhea, n (%)	21 (10.1%)	3 (3.9%)	1 (2.6%)	1 (3.9%)

WBC amounts were higher in hMPV-positive and RSV-positive individuals compared with the two other groups. NE levels in hMPV-positive individuals were higher than in the other three groups. In comparison with pH1N1 positive individuals, those infected with hMPV had increased frequency of cough. ^*^P<0.05, ^**^P<0.01. WBC, white blood cell; NE, neutrophil.

**Figure 2:**
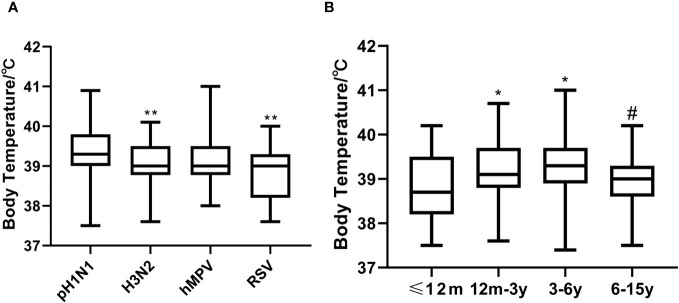
Thermal spikes among the viruses-positive groups and age groups. **(A)** Compared A (H3N2)-positive and RSV-positive individuals, pH1N1-positive patients had higher mean thermal spike (^**^ *P*<0.01). **(B)** Thermal spike differences between the ≤12m and 12m-3y groups, and the ≤12m and 3-6y groups were significant (^*^ *P*<0.05), as well as between the 12m-3y and 6-15y groups (#*P*<0.001). However, thermal spikes between the ≤12m and 6-15y groups, and the 12m-3y and 3-6y groups were not significant (all *P*>0.05).

Compared with the A (H3N2)-positive and RSV-positive individuals, pH1N1-positive patients had higher mean thermal spike (P=0.01 and <0.001, respectively) (**Figure 2A**). Children aged 12m-3y and 3-6y were more likely to have high fever compared with those aged ≤12m and 6-15y (**Figure 2B**).

## Discussion

In this study, we employed a multiplex nucleic acid assay to characterize 18 respiratory viruses or their subspecies in pediatric cases during Dec. 2018-Jan. 2019, one year previous the COVID-19 epidemic. The method had been validated before it was incorporated in the study and could detect 18 common pathogens or subtypes within 45 minutes after loading the nucleic acid sample. Based on the isothermal amplification technology and the combined MNCP platform, the assay platform had been applied to detect ILI cases with influenza virus infection during the 2017-2018 seasonal influenza epidemic, with improved performance compared with a commercially available rRT-PCR test (Li et al., 2019). After emergence of COVID-19, SARS-CoV-2 spike and nucleocapsid genes were incorporated into the system and the sequences of all the primers and probes can be found from published references (Xing et al., 2020).

From the 519 non-repeat pediatric cases, the NASBA assay revealed 430 viral infections, with a positivity rate of 82.9%. The results corroborate other studies based on rRT-PCR methods, with a positivity rate of up to 72%-95% in symptomatic ILI children, depending on age, and diagnostic and detection methods (Upadhyay et al., 2018; Busson et al., 2019; Li et al., 2019). Further analysis indicated that influenza virus A was the predominant virus (68.1%), with pH1N1 being the leading pathogen, but A (H3N2) consisted of 26.6% of Flu A positive cases. Pathogens besides influenza viruses were identified in 31.9% of the 430 cases, with hMPV at 8.8%, RSV at 6.1%, and OC43/HKU1 at 3.0% etc. The results indicate that medical professionals should be aware of pathogens other than influenza in ILI patients even during the influenza epidemic season. Given that oseltamivir therapy is widely adopted by clinicians (Lee et al., 2017), screening assays are necessary for antiviral therapy or in patients without significant improvement after oseltamivir administration. The results add value to multi-pathogen assays in pediatric clinic.

The current study also indicates significant differences in age of children infected with various pathogens. Indeed, pH1N1 was the major pathogen accounting for ILI in children of 3-6 years old, consistent with other studies showing that children in 3-5 years comprised the greatest proportion of pH1N1 cases in 2018/19 (Skowronski et al., 2019). A (H3N2) was more frequently identified in school-attending children (Wei et al., 2013). Meanwhile, hMPV was frequently detected in children between 1 and 6 years old, and RSV was detected more frequently in children younger than 3 years (Salimi et al., 2016; Thongpan et al., 2019). Only 19 cases under 1 year old included in the study, two reasons account for this, one was indeed very few baby cases present clinic with ILI in this time period, second was increased difficulty in interpreting and obtaining informed consent. We estimate that normal babies still have maternally derived antibodies that protect them from pathogenic infection (Zhang et al., 2013; Langel et al., 2022). The reasons why different ages were susceptible to variable infection might be attributed to acquired immunity such as vaccine injection, respiratory tract receptor for certain pathogen, and scope of social activities (Mansbach et al., 2021; WHO, 2022). There were 89 cases who were tested negative for pathogens listed in the study, but they could not be ruled out the possibility of mycoplasma pneumoniae or other community-source bacterial infections. In addition, all patients were sampled once, possibility of improper sampling or collection time could not be ruled out (Zhu et al., 2013; Liu et al., 2015; Zhu et al., 2019; Zhu et al., 2021).

We compared the two influenza A results of NASBA to those of the IRDT, which is not priority recommended in current guidelines but had been widely used as a POCT due to its convenience. The positivity rate (40.0%) by the IRDT was significantly lower than that obtained by the NASBA method. We compared IRDT-positive cases between the pH1N1 and A(H3N2) groups, and A(H3N2) was more frequently detected by IRDT (72.7%) than pH1N1 (27.6%). IRDT missed pH1N1 cases more than the A (H3N2) infections (P<0.01). This might be attributed to the absence of a specific antibody toward pH1N1 in the IRDT reagent. pH1N1 emerged in 2009 and replaced the sH1N1 virus as the prevalent strain. There were reports about IRDT sensitivity and its correlation with viral loads of Flu A or Flu B viruses, while our study indicates viral subtypes was a significant factor associated with sensitivity, whether it was due to viral load deserve further analysis (Li et al., 2019). The results suggested that IRDT manufacturers should keep renewing reagents even if the product has been approved for marketing. From this aspect, rRT-PCR or NASBA assays based on specific sequences may avoid this situation. Above all, the quality of swabs was fundamentally important in both the IRDT and NASBA. In the NABSA method, GAPDH was included as the internal control for swabs and RNA extraction, and the results were much more reliable than IRDT data when the reference is correct.

We analyzed clinical characteristics, including WBC, NE, fever and other symptoms among cases infected by the four viruses. The results indicated that compared with A (H3N2) -positive and RSV-positive individuals, pH1N1-positive patients were more likely to have higher temperatures. Individuals who were hMPV- and RSV-positive were more likely to present increased WBC counts compared with pH1N1-positive and A (H3N2)-positive patients (P<0.05). Children infected by hMPV tended to have higher WBC and NE counts than those infected by the other three viruses and were more likely to present cough than pH1N1-positive cases. Given that children with hMPV infection tended to get severe symptoms and were easily considered severe influenza cases or administered Oseltamivir without pathogen detection assay, attention should be paid to those not improving after antiviral therapy. Mechanism behind hMPV could cause severe diseases needs further study.

However, this study had limitations. It was a cross sectional study using random samples collected in one center from Northern China, and the sample size was relatively small, especially in those under 1 year old. In addition, the sampling time limited in 2 months, therefore, the pathogens spectrum reported in the study may be different from others (Huang et al., 2018; Yu et al., 2019). The study provides an opportunity to look at the distribution of 18 viral pathogens or their subtypes one year previous the COVID-19 epidemic. pH1N1 was the main pathogen in ILI cases in the winter of 2018-2019, but other pathogens existed as well. Other pathogens should not be neglected during the influenza season, and appropriate prevention, treatment and assay strategies are necessary for proper clinical management, in case the COVID-19 related quarantine policy being removed.

## Data availability statement

The original contributions presented in the study are included in the article/supplementary material, further inquiries can be directed to the corresponding authors.

## Ethics statement

The studies involving humans were approved by ethics committee of Beijing Tsinghua Changgung Hospital (No. 17120-0-01). The studies were conducted in accordance with the local legislation and institutional requirements. Written informed consent for participation in this study was provided by the participants’ legal guardians/next of kin.

## Author contributions

SC: Formal analysis, Investigation, Writing – original draft. YW: Methodology, Data curation, Investigation, Writing – original draft. BW: Data curation, Investigation, Writing – original draft. RL: Data curation, Writing – original draft, Software, Validation. JD: Investigation, Validation, Writing – original draft. LJ: Data curation, Writing – original draft, Methodology. XL: Investigation, Writing – original draft, Methodology. RaL: Data curation, Writing – original draft, Investigation. XY: Data curation, Investigation, Supervision, Writing – original draft. XZ: Writing – review & editing, Conceptualization, Funding acquisition, Methodology, Project administration, Resources. WL: Writing – review & editing, Supervision.
